# Novel Gyroscopic Mounting for Crystal Oscillators to Increase Short and Medium Term Stability under Highly Dynamic Conditions

**DOI:** 10.3390/s150614261

**Published:** 2015-06-17

**Authors:** Maryam Abedi, Tian Jin, Kewen Sun

**Affiliations:** 1 School of Electronics and Information Engineering, Beihang University, 37 Xueyuan Road, Haidian District, Beijing 100191, China; E-Mail: jintian@buaa.edu.cn; 2 School of Computer and Information, Hefei University of Technology, Tunxi Road 193, Hefei 230009, China; E-Mail: kewen.sun@hfut.edu.cn

**Keywords:** gyroscopic mounting, crystal oscillator, g-sensitivity, stability, high dynamic, random vibration, sinusoidal vibrations

## Abstract

In this paper, a gyroscopic mounting method for crystal oscillators to reduce the impact of dynamic loads on their output stability has been proposed. In order to prove the efficiency of this mounting approach, each dynamic load-induced instability has been analyzed in detail. A statistical study has been performed on the elevation angle of the g-sensitivity vector of Stress Compensated-cut (SC-cut) crystals. The analysis results show that the proposed gyroscopic mounting method gives good performance for host vehicle attitude changes. A phase noise improvement of 27 dB maximum and 5.7 dB on average can be achieved in the case of steady state loads, while under sinusoidal vibration conditions, the maximum and average phase noise improvement are as high as 24 dB and 7.5 dB respectively. With this gyroscopic mounting method, random vibration-induced phase noise instability is reduced 30 dB maximum and 8.7 dB on average. Good effects are apparent for crystal g-sensitivity vectors with low elevation angle φ and azimuthal angle β. under highly dynamic conditions, indicating the probability that crystal oscillator instability will be significantly reduced by using the proposed mounting approach.

## 1. Introduction

Nowadays oscillators are widely used in huge number of electronic communication, measurement and testing devices. In particular, crystal oscillators with high-Q factors are intended for use as frequency and low-phase noise standards [1]. On the other hand, crystal oscillator output stability is influenced by environmental conditions such as cleanliness and contamination, and the electromagnetic, thermal and mechanical environments [1,2,3,4,5,6,7,8,9]. Under highly dynamic conditions, crystal oscillators are exposed to a variety of dynamic loads, thus their short and medium-term stability is degraded during missions [4,5,10]. Under these conditions, the performance of the total system will be deteriorated when the short-term instability of the oscillator exceeds a specified boundary [11] determined by the host vehicle and frequency application [12].

Dynamic load-induced oscillator instability can be regarded as clock bias and drift [13]. These deviations are influenced by the magnitude and angular orientation of an inherent characteristic, *i.e.*, a g-sensitivity vector. Even among a series of carefully designed crystals with the same cut, same vibration mode and same overtone resonant frequency each crystal will have its own g-sensitivity vector. In addition, for a specified crystal, different measurement methods give different results for the magnitude and especially for the angular orientation of this vector [14]. Thus, reduction of oscillator instability is a function of an unknown parameter, which is the direction of its g-sensitivity vector. In general, two approaches are adopted to reduce the effect of dynamic loads on crystal oscillator stability [15,16]:

1-*Passive control approach*:

• Use of mechanical vibration isolation [17].
• Use of multiple, unmatched oppositely-oriented resonators [18,19].
• Reduction of resonator vibration sensitivity via resonator design [20,21,22,23,24,25].

2-*Active control approach*:

• Cancellation via feedback of accelerometer-sensed signals to the oscillator frequency tuning circuitry [16,26,27,28].

In this paper, a novel mounting method, named gyroscopic mounting, is introduced for crystal oscillators. This mounting is a self-aligned passive component, thus it does not need sensor measurement, special installation, periodical inspection, maintenance or tune ups. The most important feature is that it does not need a power supply. It senses and responds to the load and remains perpendicular to the applied load at any given moment. Furthermore, the analysis and simulations show that the proposed mounting can provide up to 30 dB improvement in phase noise that is equal to the best reduction possible with the costly and complicated accelerometer feedback method [28]. In addition, this mounting is able to suppress the attitude change of host vehicle-induced instability. In order to reduce or ideally suppress the drawbacks of the gyroscopic mounting method which could occur in the case of any dynamic load, it is proposed to use a combined method of employing the aforementioned mounting with an active vibration noise control approach using accelerometer feedback [16,26,27,28].

In this paper, all possible dynamic loads which affect the short and medium term stability of crystal oscillators under highly dynamic conditions will be introduced in Section 2. The main concept of using the gyroscopic mounting will be described in Section 3. A statistical study on the g-sensitivity vector of SC-cut crystals, which are commonly used in highly dynamic conditions, will be introduced in Section 4. In Section 5, all dynamic loads-induced instabilities and the gyroscopic mounting efficiency will be analyzed. In Section 6, the conclusions of the paper will be provided.

## 2. Dynamic Loads and Its Affection to Instability of Crystal Oscillator Output

**Figure 1:**
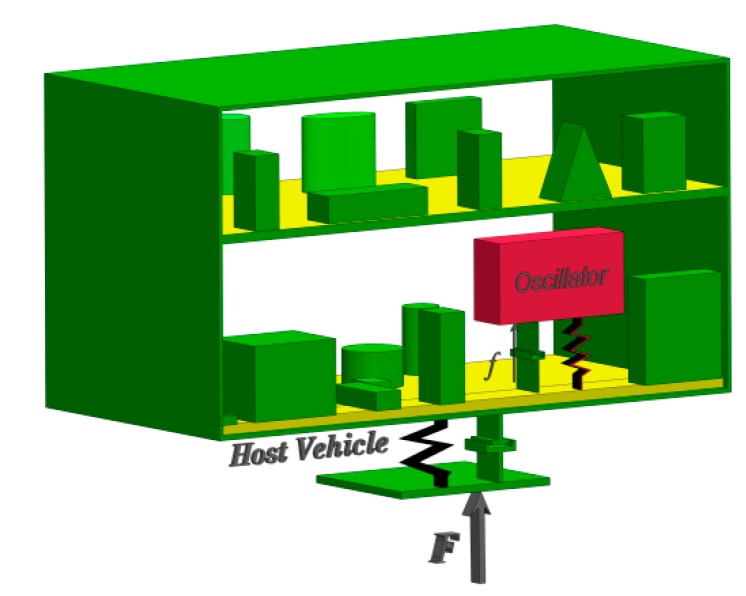
Highly dynamic host vehicle with installed crystal oscillator.

Let us assume a highly dynamic host vehicle with an installed crystal oscillator (Figure 1). Dynamic loads applied by the highly dynamic host vehicle on the crystal oscillator are divided into [7,20,21,22,23,24,25,26,27,28,29,30,31,32,33,34,35]:

1. Short-term instability sources (τ = 1 s):

• *Steady State Acceleration*: Time variant thrust of the host vehicle in both the longitudinal and lateral directions.
• *Sinusoidal Vibrations*: A series of low frequency sinusoidal vibrations.
• *Random Vibration*: A combination of band-limited sinusoidal vibrations with random amplitude, frequency and phase.

2. Medium-term instability sources (τ < 1 day):

• *Attitude and altitude changes* of the host vehicle during the mission.

*Short-term instability (Clock bias)*

(1) $$\Delta f(\text{τ}=1s)=f_{0}\vec{\Gamma }.\vec{A}=f_{0}\Gamma A\cos\text{α}\cos\text{β}$$

(2) $$\Delta \text{φ }(B_{n}=1Hz)=2\text{π}\int \Delta fdt=2\text{π}f_{0}\Gamma \int Adt$$

(3) $$£(f)=20\log\left|\frac{\Delta \text{φ}}{2}\right|$$

**Figure 2:**
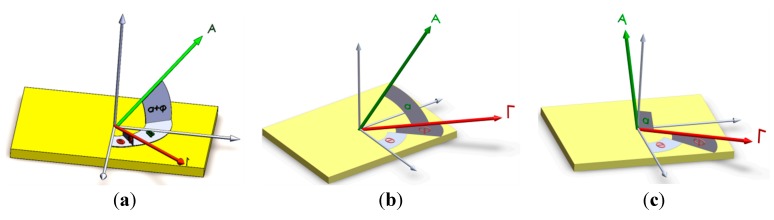
Angular orientation of load $\vec{A}$ and g-sensitivity vector $\vec{\Gamma }$ of crystal blank. (**a**) Typical state; (**b**) Critical state (β = 0); (**c**) Neutral acceleration.

*Medium-term instability (Clock drift/T)*

(4) $$\frac{\Delta f}{f_{0}}=\Gamma A\cos\text{α}\cos\text{β}$$

where Δƒ is frequency disturbance; Δφ is phase disturbance; £(ƒ) is phase noise; $\vec{\Gamma }$ is g-sensitivity vector of crystal blank (1/g) and Γ is its magnitude; φ is elevation angle of $\vec{\Gamma }$; θ is azimuthal angle of $\vec{\Gamma }$; $\vec{A}$ is the applied dynamic load (g) and A is its magnitude; α is the angle between $\vec{\Gamma }$ and $\vec{A}$; β is the angle between pages passing through $\vec{\Gamma }$ and $\vec{A}$; ƒ_0_ is oscillator frequency in Hz, T is the host vehicle mission duration. According to Equations (1)–(4) and Figure 2, frequency and phase disturbances are functions of α and β cosine. Therefore, when $\vec{A}$ moves away from $\vec{\Gamma }$, the oscillator output will be improved.

Ideally, we tend to fix the applied dynamic load perpendicular to $\vec{\Gamma }$. However, this method is not feasible in practice because each crystal blank has its own $\vec{\Gamma }$, even among crystals with the same cut, same vibration mode and same overtone resonant frequency. On the other hand, for a specified crystal, different measurement methods give different results for the magnitude and especially for the angular orientation of this vector. Also in the case of random vibration, the angular orientation of dynamic load $\vec{A}$ can be in any direction.

In this paper, the main idea to reduce these disturbances is to hold the applied dynamic load perpendicular to crystal surface in any given time instant. For a specified range of angle β shown in Figure 2 (where │β│ < cos^‒1^(sinφ/cosα)), when α is increased, the dynamic load-induced disturbances can be reduced and consequently oscillator output stability can be improved.

## 3. Gyroscopic Mounting

**Figure 3:**
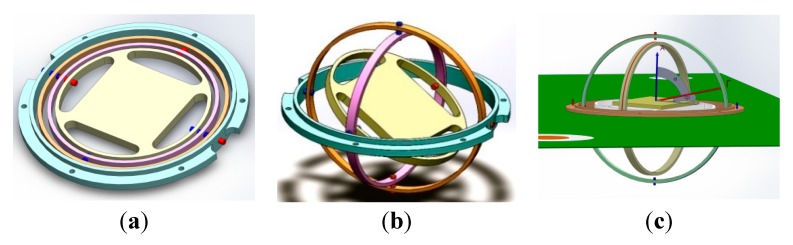
Modeling of gyroscopic mounting instrument. (**a**) Gyroscopic-mounting; (**b**) Dynamic load applied; (**c**) Mounting on PCB.

**Figure 4:**
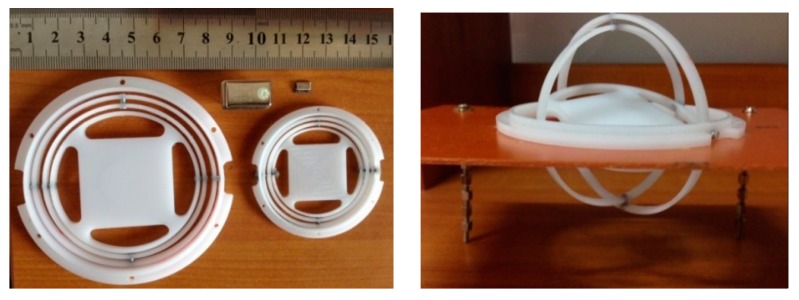
Prototype of gyroscopic mounting instrument.

In order to implement the aforementioned idea, a gyroscopic mounting method is proposed in the installation of crystal oscillator on electronic board, which gives it the freedom to rotate freely around roll, pitch and yaw. By this way, the resultant applied load will be perpendicular to oscillator surface in each time instant. Modeling and prototype of this instrument are shown in Figure 3 and Figure 4.

This mounting is a simple and cheap passive component and does not need to any power supply. It must be as quick as possible to respond to dynamic loads. We manufactured it from low-density materials and the softness of surfaces should be high as well.

After installation of the crystal oscillator on it, dynamic load-induced disturbances can be expressed as:

(5) $$\Delta f_{gyro}(\text{τ}=1s)=f_{0}\Gamma A\sin\text{φ}$$

(6) $$\Delta \varphi_{gyro}(B_{n}=1Hz)=2\text{π}\int \Delta f_{gyro}dt$$

(7) $$£{\left(f\right)}_{gyro}=20\log\left|\frac{\Delta {\text{φ}}_{gyro}}{2}\right|$$

where Δƒ_gyro_: frequency disturbance; Δφ_gyro_: phase disturbance; £_gyro_ (ƒ): phase noise. The maximum effect of gyroscopic mounting appears in the critical state shown in Figure 2b. In this case, gyroscopic mounting protects the system from the maximum probable instabilities.

## 4. G-Sensitivity Vector

**Figure 5:**
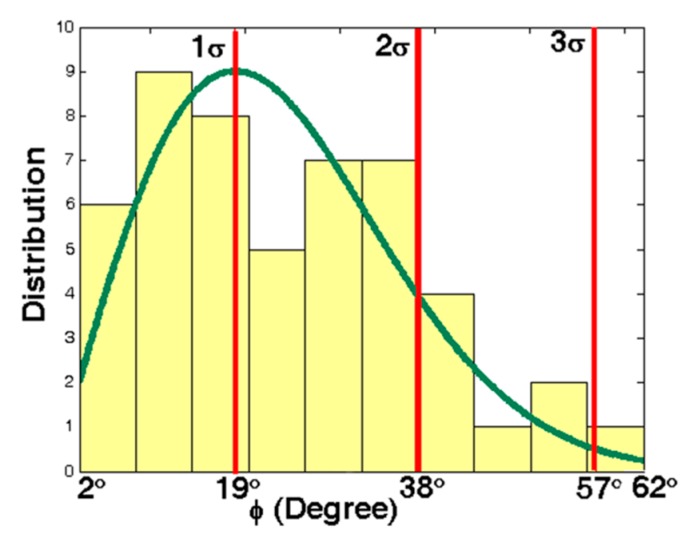
Distribution of angle |φ| for different SC-cut crystals.

Each crystal blank has its own g-sensitivity vector. Apart from its magnitude Γ, its angular orientation, especially the elevation angle φ, plays an important role on the instability induced in the oscillator output. Commonly SC-cut crystal oscillators are used under highly dynamic conditions [36]. Therefore, statistical studies have been done on the elevation angle of SC-cut crystals [25,37,38,39]. According to these studies, the angle φ follows the distribution with 1σ = 19° (42%), 2σ = 38° (84%) and 3σ = 57° (98%), φ_min_ = 2.6° and φ_max_ = 62° as shown in Figure 5.

## 5. Impacts of Dynamic Loads on Stability of Crystal Oscillator and Proof of Gyro Efficiency

Commonly GPS disciplined crystal oscillators are used as primary frequency standards for calibration and metrology in laboratories [40]. Therefore, in this paper this kind of oscillator is used to do the numerical analysis. To simulate highly dynamic conditions, the Ariane launch vehicle has been assigned as host vehicle. The parameters of this oscillator can be found in Table 1. The parameters of the Ariane launch vehicle will be mentioned in Section 5.2, Section 5.3 and Section 5.4.

**Table 1:** Characteristics of the GPS disciplined crystal oscillator.

Type		Resonant Frequency	ƒ_0_
OCXO (Oven Control Crystal Oscillator)		3rd overtone	10.23 (MHz)
**Vibration Mode**	**Crystal Cut**	${\text{σ}}_{y}$ **(Allan Deviation)**	**Γ**
Thickness shear	SC-cut	10^−11^	10^−9^/g

### 5.1. Attitude and Altitude Changes of Host Vehicle

**Figure 6:**
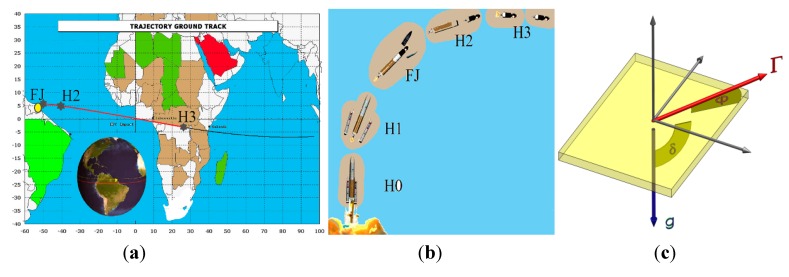
Crystal oscillator subjected to attitude and altitude changes of host vehicle. (**a**) Host vehicle trajectory ground track; (**b**) Attitude and altitude of host vehicle; Adapted from [7]. (**c**) Orientation of g, Γ and time variant angle δ on crystal.

As shown in Figure 6, the attitude of the host vehicle changes nπ (*i.e.*, 0 < δ + φ < nπ) during the mission.

On the other hand, as altitude of host vehicle increases, magnitude of gravity acceleration g decreases (g_h_ < g). Thus the angle between $\vec{\Gamma }$ and $\vec{g}$, and the magnitude of g are time variant parameters. Therefore in either case, the gravity acceleration g plays the role of dynamic load and causes disturbances in oscillator output in the form of clock bias or drift.

*Clock drift*

(8) $$\frac{\Delta f}{f_{0}}=\Gamma g_{avg}\left|\cos(\text{φ}+\text{δ})\right|\cos\text{β}$$

(9) $$\frac{g_{h}}{g}={\left(\frac{r}{r+h}\right)}^{2},g_{avg}=\frac{1}{h}\int g_{h}dh$$

**Figure 7:**
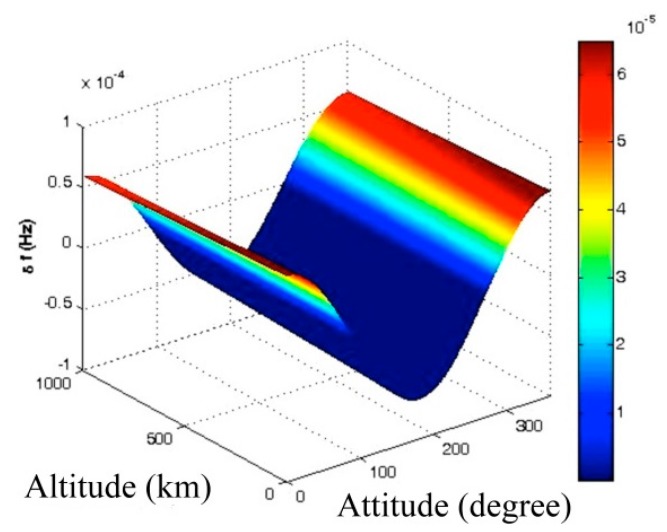
Attitude and altitude changes of host vehicle-induced clock bias.

*Note*: the altitude gravity gradient is not significant, as shown in Figure 7, therefore in calculations it has been substituted by g_avr_ on the trajectory:

*Clock bias*

(10) $$\Delta f_{gyro}(\text{τ}=1s)=f_{0}\Gamma g_{h}\cos(\text{φ}+\text{δ})\cos\text{β}$$

where: 0 < δ + φ < nπ, r = the Earth’s radius, h = altitude above the Earth’s surface, g_h_ = acceleration gravity at altitude h.

Gyro Effect

Due to the use of the gyroscopic mounting, the angle between $\vec{\Gamma }$ and $\vec{g}$, shown in Figure 6c remains constant during the mission. Therefore attitude change-induced disturbances are completely suppressed.

### 5.2. Steady State Acceleration (g)

**Figure 8:**
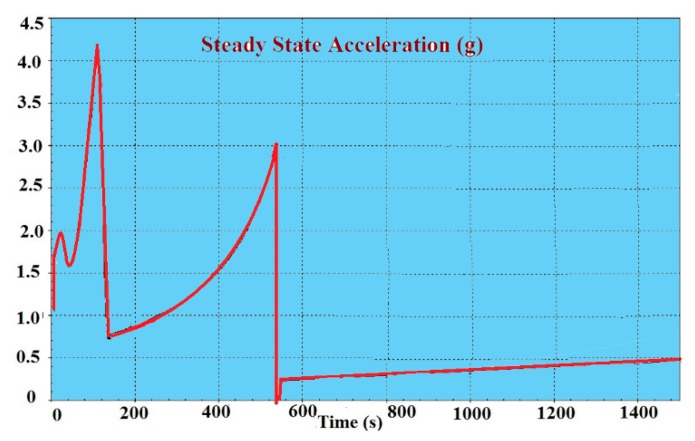
Longitudinal steady state acceleration for the Ariane 5 launch vehicle. Adapted from [7].

**Figure 9:**
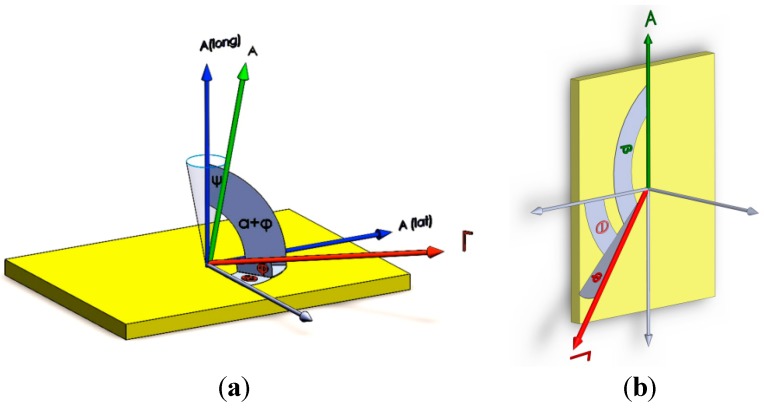
Steady state load applied on the crystal installed on the host vehicle. (**a**) Horizontally; (**b**) Vertically.

For the Ariane 5 launch vehicle, the longitudinal steady state acceleration A_long_ changes between 0 and 4.2 g on its trajectory, as shown in Figure 8, and the lateral acceleration A_lat_ changes between 0 and 0.2 g. The impact of this load on the stability of crystal oscillator output directly depends on how it is installed on the host vehicle as shown in Figure 9. To calculate the maximum gyro efficiency, analyses have been performed by assuming vertical installation.

#### 5.2.1. Impact on Crystal Oscillator Short-Term Stability

This load appears as frequency deviation and phase noise in the oscillator output and degrades its short-term stability (τ = 1*s*). These disturbances are calculated as:

*Frequency deviation, Phase Deviation and Phase Noise*

(11) $$\Delta f_{fixed-xo}(\text{τ}=1s)=f_{0}\Gamma A\cos\text{α}\cos\text{β}\;\Delta {\text{φ}}_{fixed-xo}(B_{n}=1Hz)=2\text{π}\int \Delta f_{fixed-xo}dt=2\text{π }\Delta f_{fixed-xo}\;£{\left(f\right)}_{fixed-xo}=20\log\left|\frac{\Delta {\text{φ}}_{fixed-xo}}{2}\right|$$

(12) $$\Delta f_{gyro}(\text{τ}=1s)=f_{0}\Gamma A\sin\text{φ};\Delta {\text{φ}}_{gyro}(B_{n}=1Hz)=2\text{π}\int \Delta f_{gyro}dt;£{\left(f\right)}_{gyro}=20\log\left|\frac{\Delta {\text{φ}}_{gyro}}{2}\right|$$

*Gyro Effect* on frequency and phase noise:

(13) $$f_{0}\Gamma A(\cos\text{φ}\cos\text{β}-\sin\text{φ});20\log\left|\frac{\tan\text{φ}}{\cos\text{β}}\right|$$

(14) $$\Delta f_{Allan}={\text{σ}}_{y}f_{0};\Delta {\text{φ}}_{Allan}(B_{n}=1Hz)=2\text{π }\Delta f_{Allan};£{\left(f\right)}_{Allan}=20\log\left|\frac{\Delta {\text{φ}}_{Allan}}{2}\right|$$

where: 0 < |β| < π, 0 < |α| < π; A defined in Figure 8. Critical state: β = 0.

#### 5.2.2. Results and Analysis

**Figure 10:**
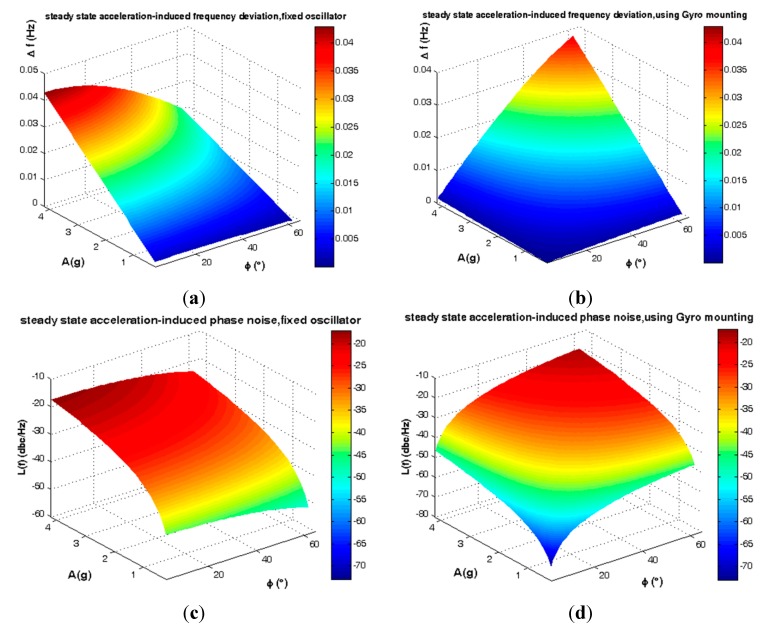
Short-term instability caused by steady state load in critical state β = 0. (**a**) Frequency deviation of fixed oscillator; (**b**) Frequency deviation of using gyroscopic mounting; (**c**) Phase noise of fixed oscillator; (**d**) Phase noise of gyroscopic mounting.

Statistically the elevation angle φ of SC-cut crystals follows the distribution of Figure 5. According to Figure 10, gyroscopic mounting shows its positive effects for the most probable crystals *i.e.*, |φ| < 38° (84%) and its drawbacks for low probability ones.

**Figure 11:**
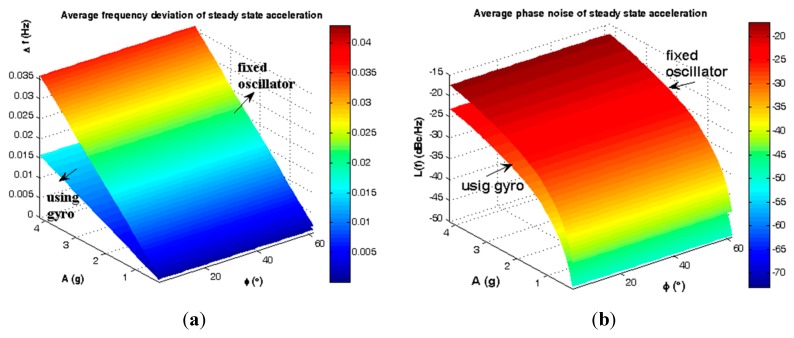
Average instability caused by steady state load. (**a**) Frequency deviation; (**b**) Phase noise.

The average steady state load-induced frequency deviation and phase noise are shown in Figure 11 and summarized in Table 2.

**Table 2:** Average efficiency of gyroscopic mounting for steady state load.

β = 0 2° < |φ| < 62°	Fixed Oscillator	Using Gyro	Gyro Effect
**Δ*ƒ_Max_***	3.59 × 10^−^^2^ (Hz)	1.57 × 10^−^^2^ (Hz)	−2.02 × 10^−^^2^ (Hz) (56.27%)
**Δ*ƒ_Min_***	1.70 × 10^−^^3^ (Hz)	7.48 × 10^−4^ (Hz)	−9.61 × 10^−4^ (Hz) (56.53%)
***£(ƒ)_Max_***	−17.42 (dBc/Hz)	−23.11 (dBc/Hz)	−5.69 (dB)
***£(ƒ)_Min_***	−43.86 (dBc/Hz)	−49.56 (dBc/Hz)	−5.69 (dB)

Where: Δ*ƒ* = Frequency deviation; *£(ƒ)* = Phase noise.

**Figure 12:**
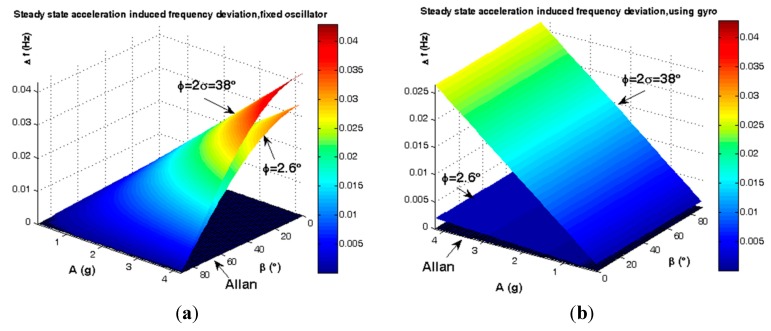
Comparison between steady state load-induced frequency deviation and Allan safety margin. (**a**) Fixed oscillator; (**b**) Gyroscopic mounting.

**Figure 13:**
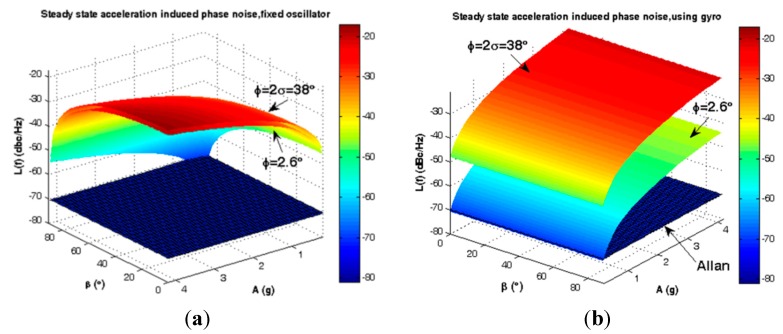
Comparison between steady state load-induced phase noise and Allan safety margin. (**a**) Fixed oscillator; (**b**) Gyroscopic mounting.

Gyroscopic mounting efficiency is as shown in Figure 12 and Figure 13 and summarized in Table 3 and Table 4 for the most probable crystals.

**Table 3:** Analysis of gyroscopic mounting efficiency for crystal with |φ| = 2.6°.

0° < β < 90°	Fixed Oscillator	Using Gyro	GYRO EFFECT
**Δ*ƒ_Max_***	4.29 × 10^−^^2^ (Hz)	1.90 × 10^−^^3^ (Hz)	−4.10 × 10^−^^2^ (Hz) (95.57%)
**Δ*ƒ_Min_***	3.57 × 10^−5^ (Hz)	9.28 × 10^−5^ (Hz)	+5.7 × 10^−5^
***£(ƒ)_Max_***	−17.40 (dBc/Hz)	−44.26 (dBc/Hz)	−26.86 (dB)
***£(ƒ)_Min_***	−79.01 (dBc/Hz)	−70.70 (dBc/Hz)	+8.31 (dB)

Where: Δ*ƒ* = Frequency deviation; *£(ƒ)* = Phase noise.

**Table 4:** Analysis of gyroscopic mounting efficiency for crystal with |φ| = 38°.

0° < β < 90°	Fixed Oscillator	Using Gyro	Gyro Effect
**Δ*ƒ_Max_***	3.39 × 10^−^^2^ (Hz)	2.65 × 10^−^^2^ (Hz)	−7.40 × 10^−^^3^ (Hz) (21.83%)
**Δ*ƒ_Min_***	2.81 × 10^−5^ (Hz)	1.30 × 10^−^^3^ (Hz)	1.27 × 10^−^^3^ (Hz)
***£(ƒ)_Max_***	−19.46 (dBc/Hz)	−21.61 (dBc/Hz)	−2.15 (dB)
***£(ƒ)_Min_***	−81.07 (dBc/Hz)	−48.05 (dBc/Hz)	33.02 (dB)

Where: Δ*ƒ* = Frequency deviation; *£(ƒ)* = Phase noise.

According to Figure 12 and Figure 13 and Table 3, for a statistically minimum φ value *i.e.*, φ = 2.6°, gyroscopic mounting has a great effect on frequency deviation which is reduced near the Allan deviation safety margin (10^−4^ Hz). Only when the β angle is very close to 90°, this mounting causes negligible drawbacks in oscillator output, such that frequency deviation is still less than the Allan deviation (10^−4^ Hz). Gyroscopic mounting shows its great effects on phase noise for β < 70°. Its drawbacks appear only for β values too close to 90°. However, this drawback is negligible because phase noise is still less than the Allan deviation (−70.05 dBc/Hz).

Based on Figure 12 and Figure 13 and Table 4, for a low elevation angle φ, gyroscopic mounting shows its positive effect on frequency deviation; however, it is still more than the Allan deviation (10^−4^ Hz). Its drawback on frequency deviation appears only for β angles too close to 90°. The best effects on phase noise appear for β < 45°. Drawbacks with small values start from β = 60°. Big drawbacks only occur for β close to 90°.

### 5.3. Sinusoidal Vibrations (f_v,si_ = 2–100 Hz)

**Figure 14:**
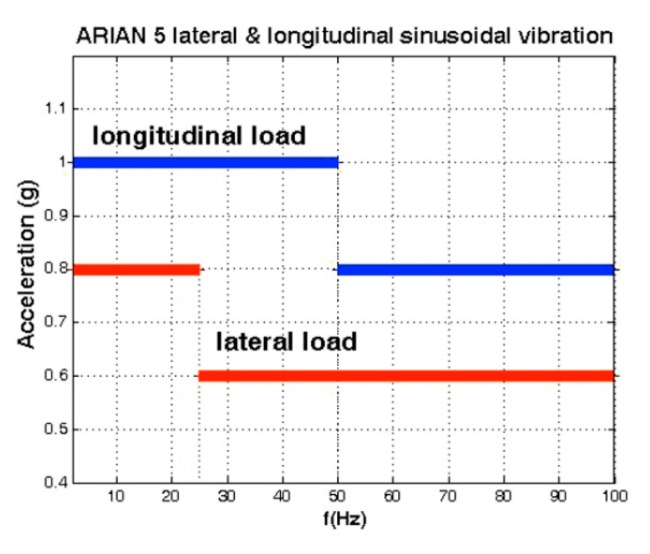
Sinusoidal vibrations of the Ariane 5 launch vehicle. Adapted from [7].

**Figure 15:**
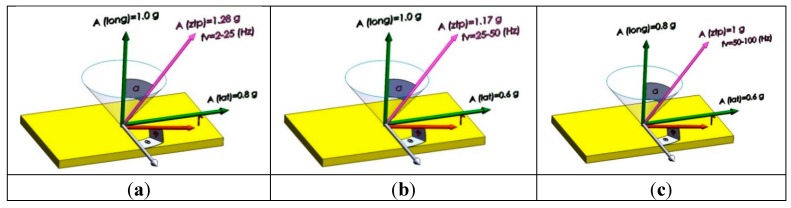
Magnitude and angular orientation of sinusoidal vibrations. (**a**) α = 38.7°; (**b**) α = 31°; (**c**) α = 36.87°.

Sinusoidal vibrations applied on the payload installed inside the fairing of Ariane 5 are as shown in Figure 14. The magnitude and angular orientation of these vibrations are illustrated in Figure 15 by dividing the frequency into three bands. That is why, in this section, two jumps are seen in the analyses figures corresponding to sinusoidal vibration-induced disturbances. The conic area in Figure 15 points out that this load can be applied on the crystal surface with any angle 0° < β < 360°, and it directly depends on the installation of the oscillator on the host vehicle.

#### 5.3.1. Impact on Crystal Oscillator Short-Term Stability

Sinusoidal vibrations appear as frequency jitter and phase noise in oscillator output and degrade its short- term stability. These disturbances are calculated as:

*Frequency Jitter, Phase jitter and Phase Noise*

$$\Delta f_{fixed-xo}(\text{τ}=1/f_{v,si})=f_{0}\Gamma A_{ztp}\sin(2\text{π}f_{v,si}t)\sin(\text{α}+\text{φ})\cos\text{β}\leq f_{0}\Gamma A_{ztp}\sin(\text{α}+\text{φ})\cos\text{β};$$

(15) $$\begin{array}{l} \Delta {\text{φ}}_{fixed-xo}(B_{n}=f_{v})=2\text{π }f_{0}\Gamma \sin(\text{α}+\text{φ})\cos\text{β }\int A_{ztp}\sin(2\text{π}f_{v,si})dt=\\ \frac{f_{0}}{f_{v,si}}{\text{A}}_{ztp}\Gamma \text{sin(α+φ) cosβ}\cos(2\text{π}f_{v,si}t)\leq \frac{\Delta f}{f_{v,si}};£{\left(f\right)}_{fixed-xo}=20\log\left|\frac{\Delta {\text{φ}}_{fixed-xo}}{2}\right| \end{array}$$

(16) $$\Delta f_{gyro}(\tau =1/f_{v})=f_{0}\Gamma A_{ztp}\sin\text{φ};\Delta {\text{φ}}_{gyro}(B_{n}=f_{v,si})=\frac{\Delta f_{gyro}}{f_{v,si}};£{\left(f\right)}_{gyro}=20\log\left|\frac{\Delta {\text{φ}}_{gyro}}{2}\right|$$

*Gyro Effect on frequency and phase noise:*

(17) $$\;f_{0}\Gamma A_{ztp}(\sin(\text{α}+\text{φ})\cos\text{β}-\sin\text{φ});20\log\left|\frac{\sin(\text{α}+\text{φ})\cos\text{β}}{\sin\text{φ}}\right|$$

(18) $$\Delta f_{Allan}={\text{σ}}_{y}f_{0};\Delta {\text{φ}}_{Allan}(B_{n}=1Hz)=\frac{\Delta f_{Allan}}{f_{v,si}};£{\left(f\right)}_{Allan}=20\log\left|\frac{\Delta {\text{φ}}_{Allan}}{2}\right|$$

where: 0 < |β| < π; 2 <ƒ_v,si_ < 100; angle α and A_ztp_ as indicated in Figure 15. Critical state and maximum effect of gyroscopic mounting appears when β = 0.

#### 5.3.2. Results and Analysis

**Figure 16:**
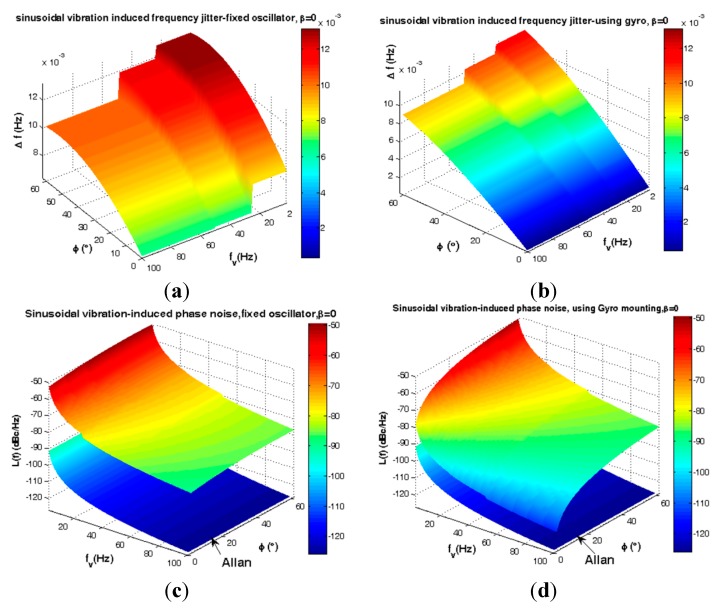
Sinusoidal vibrations-induced short term instability for critical state β = 0. (**a**) Frequency jitter of fixed oscillator; (**b**) Frequency jitter of gyroscopic mounting; (**c**) Phase noise of fixed oscillator; (**d**) Phase noise of gyroscopic mounting.

In Figure 16 the gyroscopic mounting effect on frequency jitter and phase noise is illustrated. According to this figure and Figure 5, this mounting shows its greatest advantages especially for the most probable crystals *i.e.*, |φ| < 38° (84%).

**Figure 17:**
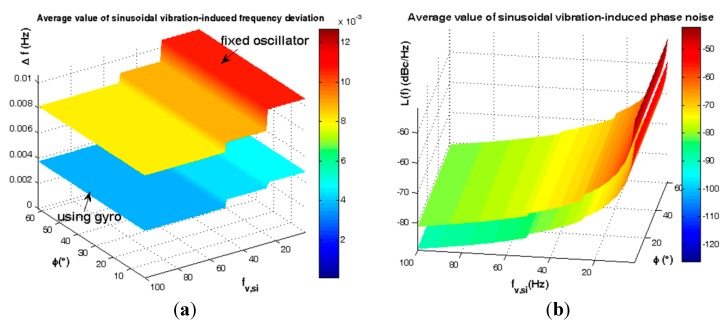
Average instability caused by sinusoidal vibration. (**a**) Frequency jitter; (**b**) Phase noise.

The average frequency jitter and phase noise are shown in Figure 17 and summarized in Table 5, with respect to the distribution specified in Figure 5 for angle φ.

**Table 5:** Average efficiency of gyroscopic mounting for sinusoidal vibration.

β = 0, 2° < |φ| < 62°	Fixed Oscillator	Using Gyro	Gyro Effect
**Δ*ƒ_Max_***	1.06 × 10^−^^2^ (Hz)	4.80 × 10^−^^3^ (Hz)	−5.80 × 10^−^^3^ (Hz) (54.72%)
**Δ*ƒ_Min_***	8.10 × 10^−^^3^ (Hz)	3.70 × 10^−^^3^ (Hz)	−4.40 × 10^−^^3^ (Hz) (54.32%)
***£(ƒ)_Max_***	−41.93 (dBc/Hz)	−49.45 (dBc/Hz)	−7.38 (dB)
***£(ƒ)_Min_***	−81.28 (dBc/Hz)	−88.66 (dBc/Hz)	−7.52 (dB)

Where: Δ*ƒ* = Frequency jitter; *£(ƒ)* = Phase noise.

**Figure 18:**
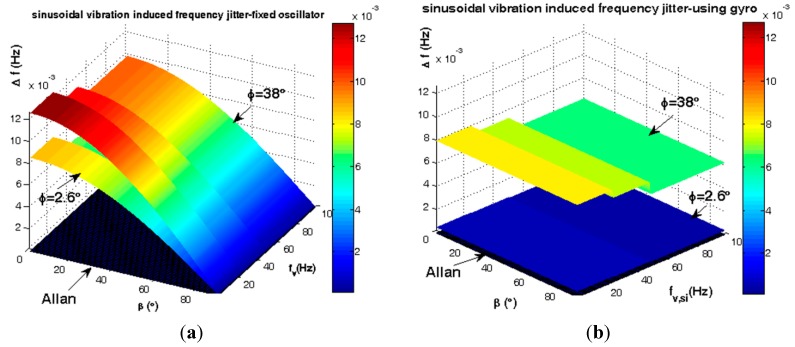
Comparison between sinusoidal vibrations-induced frequency jitter and Allan deviation safety margin. (**a**) Fixed oscillator; (**b**) Gyroscopic mounting.

**Figure 19:**
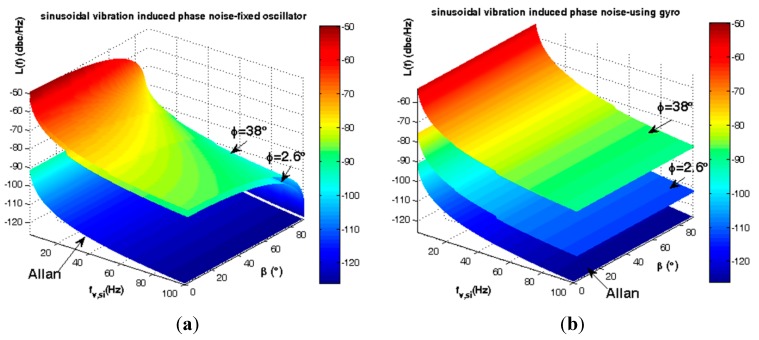
Comparison between sinusoidal vibrations-induced phase noise and Allan deviation safety margin. (**a**) Fixed oscillator; (**b**) Gyroscopic mounting.

According to Figure 5, the gyro efficiency is shown in Figure 18 and Figure 19 and summarized in Table 6 and Table 7 for elevation angle |φ| < 2σ (84%).

**Table 6:** Analysis of gyroscopic mounting efficiency for crystal with |φ| = 2.6°.

0° < β <90°	Fixed Oscillator	Using Gyro	Allan Deviation	Gyro Effect
**Δ*ƒ_max_***	8.60 × 10^−^^3^ (Hz)	5.94 × 10^−4^ (Hz)	1 × 10^−4^ (Hz)	−8.00 × 10^−^^3^ (Hz)
**Δ*ƒ_min_***	1.14 × 10^−4^ (Hz)	4.64 × 10^−4^ (Hz)	1 × 10^−4^ (Hz)	3.5 × 10^−4^ (Hz)
***£(ƒ)_min_***	−53.31 (dBc/Hz)	−76.56 (dBc/Hz)	−92.04 (dBc/Hz)	−23.25 (dB)
***£(ƒ)_min_***	−124.92 (dBc/Hz)	−112.70 (dBc/Hz)	−126.02 (dBc/Hz)	12.22 (dB)

Where: Δ*ƒ* = Frequency jitter; *£(ƒ)* = Phase noise.

**Table 7:** Analysis of gyroscopic mounting efficiency for crystal with |φ| = 38°.

0° < β < 90°	Fixed Oscillator	Using Gyro	Allan Deviation	Gyro Effect
**Δ*ƒ_max_***	1.27 × 10^−^^2^ (Hz)	8.10 × 10^−^^3^ (Hz)	1 × 10^−4^ (Hz)	−0.0047 (Hz)
**Δ*ƒ_min_***	1.72 × 10^−4^ (Hz)	6.30 × 10^−^^3^ (Hz)	1 × 10^−4^ (Hz)	0.0061 (Hz)
***£(ƒ)_min_***	−49.94 (dBc/Hz)	−53.91 (dBc/Hz)	−92.04 (dBc/Hz)	−3.98 (dB)
***£(ƒ)_max_***	−121.29 (dBc/Hz)	−90.04 (dBc/Hz)	−126.02 (dBc/Hz)	31.26 (dB)

Where: Δ*ƒ* = Frequency jitter; *£(ƒ)* = Phase noise.

According to Figure 18 and Figure 19 and Table 6, for a statistically minimum φ value *i.e.*, φ = 2.6°, gyroscopic mounting has great effect and reduces frequency jitter close to the safety margin of the Allan deviation (10^−4^ Hz). For 2 < ƒ_v,si_ < 100, this mounting reduces phase noise −23.25 dBc at most. Its drawbacks appear only when the β value is too close to 90°.

According to Figure 18 and Figure 19 and Table 7, for 2 < ƒ_v,si_ < 100, gyroscopic mounting reduces the phase noise −3.97 dBc at most. The best effects on phase noise appear for β < 45°. Small drawbacks start from β = 60° and big ones only occur for β values close to 90°.

### 5.4. Random Vibration (20 < ƒ_RV_ < 2000 Hz)

**Figure 20:**
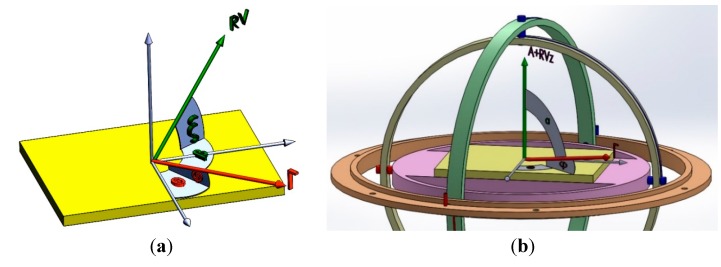
Random vibration applied on crystal oscillator 0 < |ξ| < π, 0 < |β| < π. (**a**) Fixed oscillator; (**b**) Oscillator on gyroscopic mounting.

Random vibration is a band-limited noise with Gaussian distribution and could be created by different sources and applied in any direction *i.e.*, 0 < |ξ| < π and 0 < |β| < π as shown in Figure 20a.

**Figure 21:**
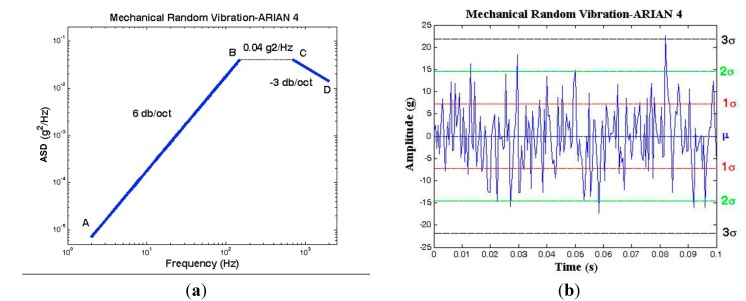
Mechanical random vibration for the Ariane 4 launch vehicle. (**a**) ASD (g^2^/Hz); Adapted from [32]. (**b**) Time domain representation.

Mechanical random vibration of the Ariane 4 launch vehicle is shown in Figure 20a [32]. Generally, this load is defined by Acceleration Spectral Density (ASD) in (g^2^/Hz). Since random vibration is a combination of all the frequencies at the same time, it is necessary to configure this load in the time domain. According to the Parseval’s law, g_rms_ is equal to 1σ of random vibration. Thus the random vibration in time domain can be shown according to Figure 21b by calculation of g_rms_ from ASD.

On the other hand, there could be an infinite number of input ASD curves which have the same area, and therefore the same g_rms_. Therefore, beside analysis in time domain, it is necessary to analyze the random vibration in the frequency domain.

#### 5.4.1. Random Vibration in Time Domain

*(a) Calculation of g_rms_*

(19) $$g_{rms}=\sqrt{ASD\times f}$$

(20) $$From\;point\;A\;to\;B:20<f<150,ASD=1.7778\times 10^{-6}f^{2}.$$

(21) From point B to C:150≤f<700,ASD=0.04.

(22) $$From\;point\;C\;to\;D:700\leq f<2000,ASD=\frac{28}{f}.$$

(23) $$g_{rms}(1\text{σ})=7.3g$$

(b) Impact on crystal oscillator short-term stability

Random vibration causes short-term instability as frequency jitter and phase noise in oscillator output.

*Frequency jitter, Phase jitter and Phase Noise*

(24) $$\Delta f_{fixed-xo}(\text{τ}=\frac{1}{f_{RV}})=f_{0}\Gamma A\cos(\text{ξ}-\text{φ})\cos\text{β};\;\Delta {\text{φ}}_{fixed-xo}(B_{n}=f_{v})=2\text{π}f_{0}\Gamma A\cos(\text{ξ}-\text{φ})\cos\text{β}\int \sin(2\text{π}f_{vi}t)dt=\frac{\Delta f}{f_{RV}};\;£{\left(f\right)}_{fixed-xo}=20\log\left|\frac{\Delta {\text{φ}}_{fixed-xo}}{2}\right|$$

(25) $$\Delta f_{gyro}(\text{τ}=\frac{1}{f_{RV}})=f_{0}\Gamma A\sin\text{φ};\Delta {\text{φ}}_{gyro}(B_{n}=f_{RV})=\frac{\Delta f_{gyro}}{f_{RV}};£{\left(f\right)}_{gyro}=20\log\left|\frac{\Delta {\text{φ}}_{gyro}}{2}\right|$$

*Gyro Effect on frequency and phase*

(26) $$f_{0}\Gamma A(\cos(\text{ξ}-\text{φ})\cos\text{β}-\sin\text{φ});20\log(\frac{\cos(\text{ξ}-\text{φ})\cos\text{β}}{\sin\text{φ}})$$

(27) $$\Delta f_{Allan}={\text{σ}}_{y}f_{0};\Delta {\text{φ}}_{Allan}(B_{n}=1Hz)=\frac{\Delta f_{Allan}}{f_{RV}};£{\left(f\right)}_{Allan}=20\log\left|\frac{\Delta {\text{φ}}_{Allan}}{2}\right|$$

where: Critical state is β = 0 and ξ = φ. Maximum gyro effect appears when β = kπ and ξ − φ = kπ (k = 0, 1 …).

(c) Results and Analysis

To analyze the effects of gyroscopic mounting on random vibration, it is necessary to determine three unknown parameters; angles ξ, β and φ, as shown in Figure 20.

**Figure 22:**
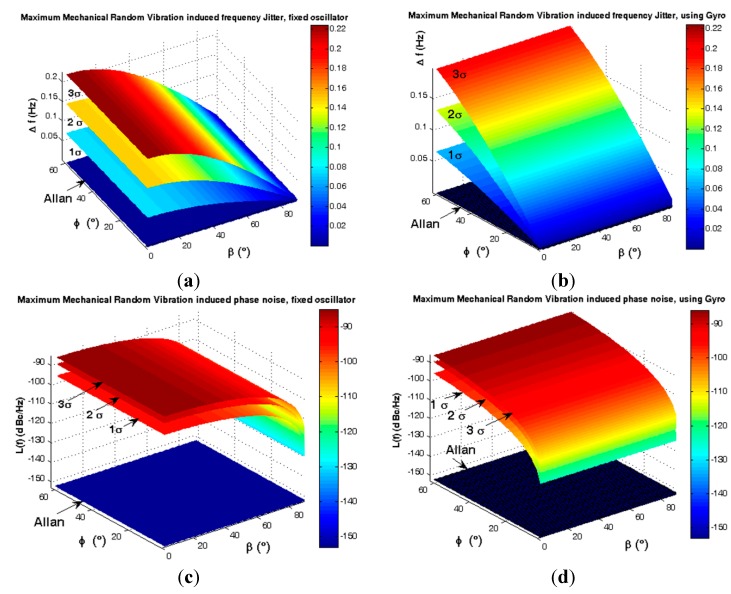
Maximum instability (ξ = φ) caused by random vibration for ƒ_RV_ = 2000 (Hz). (**a**) Frequency jitter of fixed oscillator; (**b**) Frequency jitter of gyroscopic mounting; (**c**) Phase noise of fixed oscillator; (**d**) Phase noise of gyroscopic mounting.

**Figure 23:**
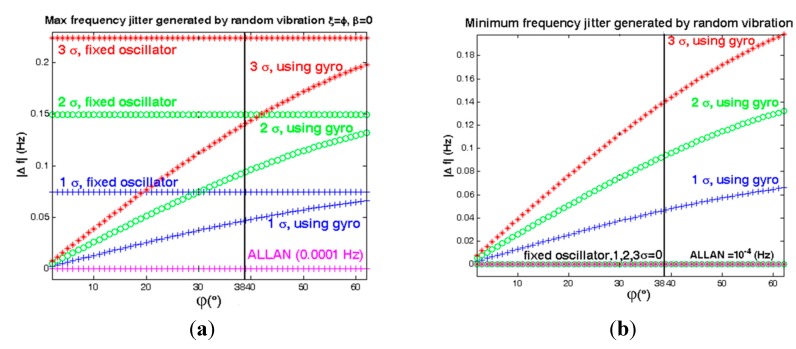
Comparison between system instability in different states. (**a**) Maximum state ξ = φ, β = 0; (**b**) Minimum state, ξ − φ = 90°or β = 90°.

If ξ = φ and β = 0, thus $\vec{\Gamma }$ and $\vec{A}$ coincide each other, therefore this mounting shows the best positive effects for low elevation angle φ. If ξ – φ = π/2 or β = π/2, then $\vec{\Gamma }$ and $\vec{A}$ are perpendicular to each other, in this case gyroscopic mounting shows the worst performance. These results are shown in Figure 22 and Figure 23.

**Figure 24:**
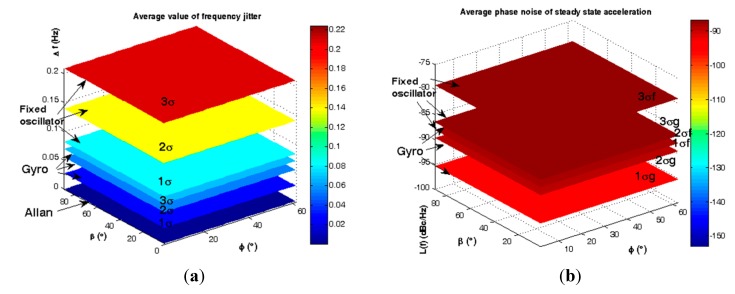
Average instability caused by random vibration. (**a**) Frequency jitter; (**b**) Phase noise.

According to Figure 22 and Figure 23 and Table 8, the maximum effects of gyroscopic mounting on frequency jitter can be obtained when β = 0 and φ = 2.6°. In this case, gyroscopic mounting reduces the frequency jitter to near the safety margin of the Allan deviation. Maximum effect on phase noise appears for β = 0 and φ = 2° and this positive effect continues up to β = 60°. Drawbacks start from β > 60° and its maximum value occurs for β = 90° and φ = 38°. With regard to the distribution shown in Figure 5 for elevation angle φ, the average frequency jitter and phase noise are as shown in Figure 24 and summarized in Table 9.

**Table 8:** Maximum effects and drawbacks of gyroscopic mounting on random vibration.

Random Vibration Induced Instability				
2.6° < φ < 38°, RV = 3σ, ξ = φ, β = 0	Fixed Oscillator	Using Gyro	Allan	Gyro Effect
***Max gyro effect on* Δ*ƒ***	2.24 × 10^−^^1^ (Hz)	7.80 × 10^−^^3^ (Hz)	1 × 10^−4^ (Hz)	−2.16 × 10^−^^1^ (Hz) (96.42%)
***Max gyro drawback on* Δ*ƒ***	3.90 × 10^−^^3^ (Hz)	1.14 × 10^−^^1^ (Hz)	1 × 10^−4^ (Hz)	+1.10 × 10^−^^1^ (Hz)
***Max gyro effect on £(ƒ)***	−85.03 (dBc/Hz)	−114.17 (dBc/Hz)	−152.04(dBc/Hz)	−29.14 (dB)
***Max gyro drawback on £(ƒ)***	−120.19 (dBc/Hz)	−91.31 (dBc/Hz)	−152.04(dBc/Hz)	+28.88 (dB)

Where: Δ*ƒ* = Frequency jitter; *£(ƒ)* = Phase noise.

**Table 9:** Average efficiency of gyroscopic mounting for random vibration.

β = 0, 2° < |φ| < 62°		Fixed Oscillator	Using Gyro	Gyro Effect
**Δ*ƒ***	**1σ (68.3%)**	0.0697 (Hz)	0.0273 (Hz)	0.0424 (Hz)
	**2σ (95.6%)**	0.1394 (Hz)	0.0546 (Hz)	0.0847 (Hz)
	**3σ (99.7%)**	0.2091 (Hz)	0.0819 (Hz)	0.1271 (Hz)
***£(ƒ)***	**1σ (68.3%)**	−88.16 (dBc/Hz)	−96.89 (dBc/Hz)	−8.73 (dB)
	**2σ (95.6%)**	−82.55 (dBc/Hz)	−91.28 (dBc/Hz)	−8.73 (dB)
	**3σ (99.7%)**	−79.27 (dBc/Hz)	−87.99 (dBc/Hz)	−8.73 (dB)

#### 5.4.2. Random Vibration in Frequency Domain

*(a) Calculations*

(28) $$RV_{\max}=\sqrt{2}g_{rms}=\sqrt{2ASD\times f}$$

(29) $$From\;point\;A\;to\;B:20<f<150;RV=0.0019\times f^{1.5}(g).$$

(30) $$From\;point\;B\;to\;C:150\leq f<700;RV=0.2828\times f^{0.5}(g).$$

(31) From point C to D:700≤f<2000;RV=7.4833(g).

(b) Impact on crystal oscillator short**-**term stability

*Frequency jitter, Phase Jitter and Phase Noise*

(32) $$\Delta f_{fixed-xo}(\text{τ}=\frac{1}{f_{v}})=f_{0}\Gamma RV\sin(2\text{π}f_{v}t)\cos(\text{ξ}-\text{φ})\cos\text{β};\;\Delta {\text{φ}}_{fixed-xo}(B_{n}=f_{v})=2\text{π}f_{0}\Gamma \cos(\text{ξ}-\text{φ})\cos\text{β}RV\int \sin(2\text{π}f_{v}t)dt\leq \frac{\Delta f}{f_{v}}\;£{\left(f\right)}_{fixed-xo}=20\log\left|\frac{\Delta {\text{φ}}_{fixed-xo}}{2}\right|$$

(33) $$\Delta f_{gyro}(\text{τ}=\frac{1}{f_{v}})=f_{0}\Gamma RV\sin(2\text{π}f_{v}t)\sin\text{φ};\Delta {\text{φ}}_{gyro}(B_{n}=f_{v})=\frac{\Delta f_{gyro}}{f_{v}};£{\left(f\right)}_{gyro}=20\log\left|\frac{\Delta {\text{φ}}_{gyro}}{2}\right|$$

*Gyro Effect on frequency and phase*

(34) $$f_{0}\Gamma RV(\cos(\text{ξ}-\text{φ})\cos\text{β}-\sin\text{φ});20\log|\frac{\cos(\text{ξ}-\text{φ})\cos\text{β}}{\sin\text{φ}}|;$$

(35) $$\Delta f_{Allan}={\text{σ}}_{y}f_{0};\Delta {\text{φ}}_{Allan}(B_{n}=1Hz)=\frac{\Delta f_{Allan}}{f_{v}};£{\left(f\right)}_{Allan}=20\log\left|\frac{\Delta {\text{φ}}_{Allan}}{2}\right|$$

where: 20 < ƒ_v_ < 2000 (Hz), RV: according to Equations (28)–(31), 0 < |β| < π, 0 < |ξ| < π, 0 < |φ| < π/2, Critical state or maximum gyroscopic mounting effect appears when: β = kπ & ξ − φ = kπ (k = 0, 1).

(c) Results and Analysis

**Figure 25:**
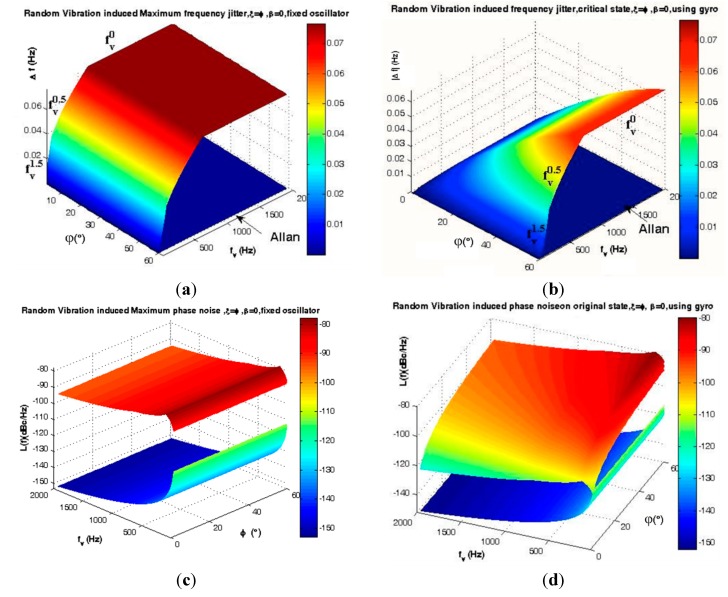
Analysis of random vibration in frequency domain for ξ = φ, β = 0. (**a**) Frequency jitter of fixed oscillator; (**b**) Frequency jitter of gyroscopic mounting; (**c**) Phase noise of fixed oscillator; (**d**) Phase noise of gyroscopic mounting.

According to Figure 20, when random vibration is applied in the same direction as the g-sensitivity vector, *i.e.*, $\vec{\Gamma }$ and $\vec{A}$ coincide each other and therefore ξ = φ and β = 0, the maximum instability induces to oscillator output which is shown in Figure 25a,c. In this case, the gyroscopic mounting presents its maximum effect as shown in Figure 25b,d. This positive effect is in the maximum state for low elevation angle φ.

**Figure 26:**
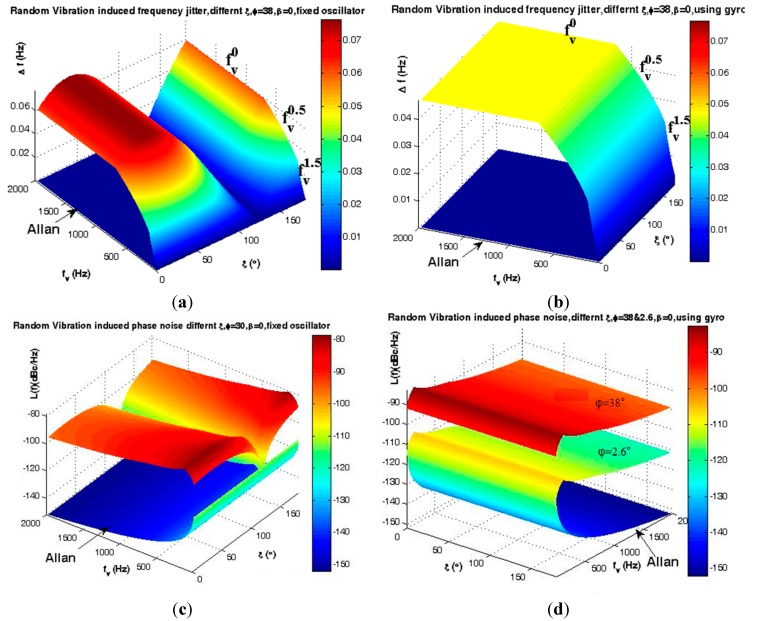
Analysis of random vibration in frequency domain for 0° < |ξ | < 180°, β = 0, φ = 2σ = 38°.(**a**) Frequency jitter of fixed oscillator; (**b**) Frequency jitter of gyroscopic mounting; (**c**) Phase noise of fixed oscillator; (**d**) Phase noise of gyroscopic mounting.

According to Figure 20, in practice random vibration could be applied in any direction *i.e.*, 0° < |ξ| < 180°. In this case, the dynamic load induces instability in the oscillator output, which is shown in Figure 26a,c and the gyroscopic mounting presents its maximum effect, which is shown in Figure 26b,d. In this case, when ξ − φ = 90° or β = 90°, gyroscopic mounting induces its maximum drawback which is limited to the angles close to the specified margin of ξ − φ = 90°. As shown in Figure 26d, better effects appear for low elevation angle φ.

## 6. Conclusions

According to the analysis results, gyroscopic mounting protects systems from instabilities caused by dynamic loads. It shows benefits on reduction of clock drift caused by the attitude change of the host vehicle. The mounting can also reduce the disturbances induced by steady state acceleration. The results of 27 dB maximum and 5.7 dB on average in phase noise improvement have been validated in our analyses. When considering sinusoidal vibrations, gyroscopic mounting reduces disturbances close to the safety margin of the Allan deviation. The phase noise improvement is 24 dB maximum and 7.5 dB on average. The random vibration-induced instability can be reduced 30 dB maximum and 8.7 dB on average in phase noise. The gyroscopic mounting for crystal oscillators has been proven to be able to improve the frequency deviation and phase noise caused by dynamic loads. It is promising for use in low cost frequency standard improvement for civil aviation and military purposes.
